# Differing views: Can chimpanzees do Level 2 perspective-taking?

**DOI:** 10.1007/s10071-016-0956-7

**Published:** 2016-02-06

**Authors:** Katja Karg, Martin Schmelz, Josep Call, Michael Tomasello

**Affiliations:** Department of Psychology, Max Planck Institute for Evolutionary Anthropology, Leipzig, Germany; School of Psychology and Neuroscience, University of St Andrews, St Andrews, UK

**Keywords:** Perspective-taking, Appearance–reality, Deception, False belief, Chimpanzee

## Abstract

Although chimpanzees understand *what* others may see, it is unclear whether they understand *how* others see things (Level 2 perspective-taking). We investigated whether chimpanzees can predict the behavior of a conspecific which is holding a mistaken perspective that differs from their own. The subject competed with a conspecific over two food sticks. While the subject could see that both were the same size, to the competitor one appeared bigger than the other. In a previously established game, the competitor chose one stick in private first and the subject chose thereafter, without knowing which of the sticks was gone. Chimpanzees and 6-year-old children chose the ‘riskier’ stick (that looked bigger to the competitor) significantly less in the game than in a nonsocial control. Children chose randomly in the control, thus showing Level 2 perspective-taking skills; in contrast, chimpanzees had a preference for the ‘riskier’ stick here, rendering it possible that they attributed their own preference to the competitor to predict her choice. We thus run a follow-up in which chimpanzees did not have a preference in the control. Now, they also chose randomly in the game. We conclude that chimpanzees solved the task by attributing their own preference to the other, while children truly understood the other’s mistaken perspective.

## Introduction

Chimpanzees are proficient when judging what others can see—they follow others’ gaze direction (Tomasello et al. 1998), even around barriers (Bräuer et al. 2005; Hare et al. 2000), can take into account what a competitor can and cannot see when competing for food (Bräuer et al. 2007; Hare et al. 2000; Kaminski et al. 2008), and make use of what a competitor has seen in the past (Hare et al. 2001; MacLean and Hare 2012). They thus know that their perspective can differ from the perspective of others in the sense that they know that sometimes they can see objects that others cannot see and vice versa.

While it is undisputed that chimpanzees possess these so-called Level 1 perspective-taking skills (Flavell et al. 1978, 1981), it is unclear whether they also have a deeper understanding of the other’s perspective. Flavell and colleagues (Flavell et al. 1978, 1981) introduced the concept of Level 2 perspective-taking to describe higher-level understanding of perspectives—an understanding that the same object might appear differently from another perspective (Flavell et al. 1981). A classic way to test Level 2 perspective-taking in children is to place a picture of a turtle between the experimenter and the child, and ask whether it is standing on its feet or lying on its back from the experimenter’s perspective (Flavell et al. 1981; Masangkay et al. 1974). There is evidence that in humans, Level 1 perspective-taking is mastered by the age of 2 years (Flavell et al. 1978; McGuigan and Doherty 2002; Moll and Tomasello 2006), but Level 2 perspective-taking takes longer, until age 3 (Moll and Meltzoff 2011), with many studies suggesting an even later emergence (Flavell et al. 1980, 1981; Masangkay et al. 1974; Pillow and Flavell 1986).

Although Level 2 perspective-taking is harder than Level 1, it is conceivable that chimpanzees may understand that the same object might appear differently from another’s perspective. Nonsocial appearance–reality tasks show that great apes know that their own perspective does not always reflect reality (Karg et al. 2014; Krachun et al. 2009). For example, Karg et al. (2014) presented great apes one large and one small edible bread stick and occluded them such that the size relations seemed reversed. Subjects could then choose which one they wanted. When subjects had seen the real stick sizes before, they disregarded the apparent (deceptive) stick sizes and chose according to the real size relations, not according to their current perception. They did not do so in a control condition in which they were naïve about the true size relations. In a similar study with chimpanzees, Krachun et al. (2009) excluded object tracking as an explanation for the results.

Chimpanzees thus seem to understand that their own perspective can differ from reality. However, do they also understand that others can hold a mistaken perspective? If they do, they should be able to use this for deceiving others. However, evidence for chimpanzees’ ability to use deception to create a false belief in others is limited. There are several anecdotes that chimpanzees deceive others by hiding themselves or body parts from their view (de Waal 1998; Whiten and Byrne 1988), and experimental studies support this notion (Hare et al. 2006; Melis et al. 2006). Yet, experiments have found that chimpanzees’ deceptive skills have limited flexibility, raising the question of whether chimpanzees deceive and hide because they really understand the other’s false belief, or rather because they have learned rules about the relation of others’ line of sight and their behavior in the past (Anderson 1986; Heyes 1993, 1998).

Another line of research casts doubt on whether chimpanzees are able to understand that another individual can hold a mistaken perspective: research on false-belief understanding. While human children succeed in implicit tasks by the age of about 1 year (for a review, see Baillargeon et al. 2010) and in explicit tasks by the age of 4 years (Wellman et al. 2001; Wimmer and Perner 1983), chimpanzees consistently fail in these tasks, although several different paradigms have been used (Call and Tomasello 1999; Kaminski et al. 2008; Krachun et al. 2009, 2010; O’Connell and Dunbar 2003).

Thus, on the one hand, chimpanzees have great difficulties ascribing representations to others that differ from reality and are independent of the other’s current perception (as in false-belief scenarios), while on the other hand they are highly proficient in Level 1 perspective-taking tasks. We were thus interested in whether they would succeed on an intermediate level—ascribing false perceptions, not false belief, to others. We investigated whether chimpanzees can predict a conspecific’s false perspective that differed from their own and from reality. The subject sat opposite a conspecific competitor, with a vertical board between them. On the subject’s side, two same-sized edible sticks were attached to the board, one reaching over the edge of the board more than the other, such that it seemed bigger from the competitor’s perspective. The subject could see that both sticks had the same size. In a previously established competitive game (see Kaminski et al. 2008; Schmelz et al. 2011, 2013), the competitor could now choose one of the sticks in private, and the subject chose thereafter, without knowing which of the two sticks was gone. We predicted that if subjects understood the other’s mistaken perspective, they would prefer the stick that looked smaller from the competitor’s side in the social condition, but would not do so in a nonsocial control condition, in which there was no competitor present.

## Study 1: chimpanzees and children

### Method

#### Subjects

Sixteen chimpanzees (*Pan troglodytes*) participated as subjects in this experiment, 11 females and five males ranging in age from 7 to 36 years (*M* = 21.1 years). Eight apes were nursery-reared, whereas eight were mother-reared. Two chimpanzees were excluded due to uncooperativeness in the pretest (Annett, 12, and Corry, 35). In addition, two chimpanzees served as competitors; they were Kara (female, mother-reared, 7) for the A chimpanzee group, and Gertruida (female, mother-reared, 19) for the B chimpanzee group. The competitors never switched roles with the subjects and vice versa. All subjects were housed at the Wolfgang Köhler Primate Research Center at the Leipzig Zoo (Germany), where they lived in social groups and had access to indoor and outdoor areas. Subjects were tested in their indoor enclosures, fed according to their daily routine and never food- or water-deprived. Subjects all had previous experience with participating in experimental studies and with the general procedure of the competitive game from previous studies (Kaminski et al. 2008; Schmelz et al. 2011, 2013).

Sixteen 6-year-old children also participated in this experiment, six boys and 10 girls ranging in age from 6/0 to 6/5 (year/months). Six more children participated, but were not included in the final sample because they did not pass the pretest (5) or were not present at their second testing day (1). Children were recruited from kindergartens in Leipzig (Germany). They were not informed of the purpose of the study and were encouraged to collect as many bread sticks as possible, preferably the big ones. An adult stooge served as their competitor.

#### Materials

The setup and procedure were based on the previously successfully applied ‘chimp chess’ method by Kaminski et al. (2008).

##### Chimpanzees

**Fig. 1:**
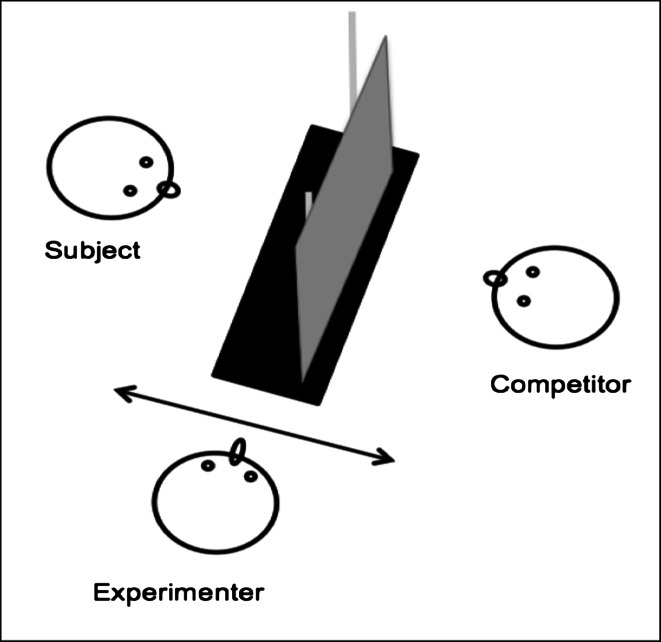
Schematic bird’s eye view of the experimental setup

A sliding Table (20 × 80 cm) was placed on a platform (82.5 × 80 cm) between two enclosures, such that the subject and the competitor could sit opposite to each other and the experimenter could move the sliding table back and forth between them (Fig. 1). The two enclosures adjoined each other, and chimpanzees could see each other through the mesh parts that were not blocked by our setup (e.g., they could look through the mesh next to or underneath the apparatus). As the chimpanzees moved around freely in their enclosures, we are thus confident that they were aware of the presence of the competitor in the social condition.

**Fig. 2:**
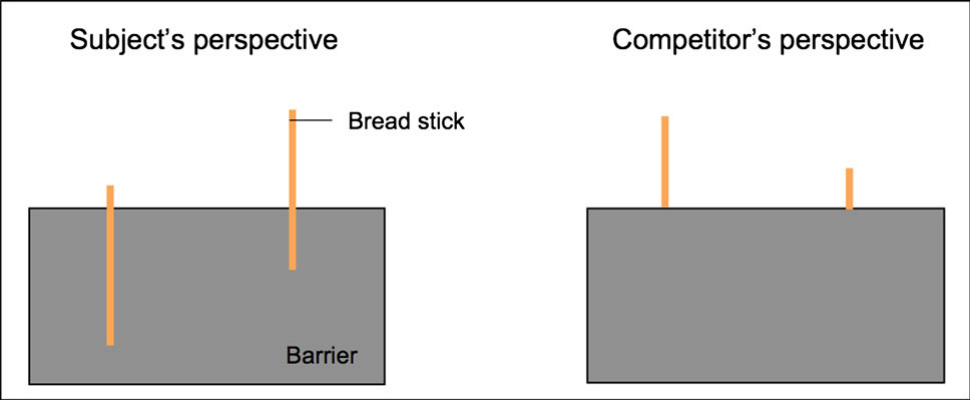
Subject’s and competitor’s view of the experimental setup

A barrier was placed on the table between the subject and the competitor. Depending on the phase of the experiment, a transparent barrier (44 × 35.5 cm) or an opaque barrier (44 × 21 cm) was used. Two bread sticks (equally sized in the test, or one half as long as the other in the pretest and training) could be fixed to the subject’s side of the barrier with an elastic strap, such that in the test the subject could see they had the same length, whereas it seemed like one was much bigger than the other to the competitor (Fig. 2). A sliding door (55 × 96 cm) could be installed on the subject’s side of the sliding table to prevent the subject from seeing the sticks. The subject could slide it open to the left or right side and could then see the stick on the opened side (their view on the other stick was blocked by a small vertical barrier between the two sticks). An additional red occluder (80 × 51 cm) was used to block the competitor’s or subject’s view when necessary. Chimpanzees could choose a stick by poking their fingers through the corresponding side of the mesh, or, if the door was installed, subjects could choose by sliding the door open (they would receive the stick that they revealed, if one was present).

##### Children

The setup was the same as for the chimpanzees, except that they sat at a table in their kindergartens, and their competitor was an adult stooge. Children received ‘treasure boxes’ and were told that the goal of the game was to collect as many sticks as possible, preferably the big ones.

#### Design

Each subject was tested in a social and a nonsocial condition. Chimpanzees received two consecutive sessions with 12 trials each per condition, resulting in 24 trials in total. Due to testing time constraints, children received one session with eight trials per condition. Sessions were administered on different days, with a maximum of 4 days between sessions. The order of conditions was counterbalanced for sex (children) and age and sex (chimpanzees).

#### Procedure

The procedure was the same for chimpanzees and children, except that chimpanzees had to pass a procedure training before receiving test sessions, to ensure that they (1) knew how to operate the sliding door, and (2) understood the course of events. The procedure training trials were the same as in the social pretest (see below), and subjects had to choose correctly in at least 10/12 trials in two consecutive sessions by choosing the remaining smaller stick after the competitor.

Since children had never played chimp chess, they received verbal instructions and a demonstration instead. Before each session started, the experimenter briefly explained the game to them. She installed two big bread sticks at the transparent barrier on the sliding table and slid the table back and forth on the table. She explained that the players were allowed to choose one of the bread sticks by pointing to them when the barrier was on their side. She then introduced the sliding door, slid the table to the subject’s side, let the subject open the door on both sides once, and gave her the corresponding stick.

After the chimpanzees passed the procedure training and the children experienced the door demonstrations, all subjects moved on to the test sessions. The procedure was the same in the two test conditions, except that there was no competitor present in the nonsocial condition.

Each session consisted of three phases: pretest (8 trials), training (8 trials), and test (12 trials). In each phase, the experimenter started baiting the string on her far side first and the other thereafter according to a predefined scheme that determined which of the sticks was the bigger-looking one from the competitor’s side (or the truly bigger one in the pretest and training). The stick constellation order was randomized with the constraint that the same constellation of bread sticks could not be presented more than twice in a row.

1. *Pretest* The transparent barrier was placed on the centered sliding table, and a big and a small bread stick were attached to the strings (with their upper ends aligned). Then, the table was slid to the competitor’s side, the competitor chose the longer stick by sticking her finger through the corresponding side of the mesh (nothing happened in the nonsocial condition), the sliding door was installed, and the table was moved to the subject. Now, the subject was allowed to open the door to one side and receive the reward behind it (big or small stick in the nonsocial condition, small or no stick in the social condition). The pretest ensured that the subject paid attention to the general course of events, and that she remembered the location of the remaining stick. Subjects were only included in the final sample if they chose the remaining stick reliably (at least seven out of eight trials) after having seen the competitor choosing. In addition, they had to demonstrate that they had understood the goal of the game by showing a preference for the bigger stick in at least six out of eight trials when choosing between a big and a small stick in the nonsocial condition.
2. *Training* The procedure was similar to the pretest, but with the following changes: The additional red barrier was introduced to ensure that the players could never see each other, thus excluding the possibility that the subject could base her decision on subtle cues like the competitor’s gaze direction instead of mentally representing her perspective. The barrier was placed in front of the competitor’s viewing window during the baiting (with no competitor present in the nonsocial sessions) and subsequently was placed in front of the viewing window of the player while the competitor was choosing. In addition, the sliding door was installed right after the baiting. This training ensured that the subjects got used to the procedure and, in the social sessions, to the fact that after the competitor had chosen, there was only one stick (the smaller) left (while in the nonsocial session, both sticks were still present).
3. *Test* The opaque barrier was placed on the sliding table, and the competitor’s viewing window was blocked. Two big bread sticks of the same size were attached to the barrier on the subject’s side, so that from the competitor’s side, one stick looked longer than the other (Fig. 2). The procedure was the same as in the training, except that the competitor did not receive any of the sticks after choosing (for the chimpanzees, the competitor secretly received a reward from the experimenter, to keep up her attention; in the children’s case, the adult stooge just pretended to put a stick in her box by noisily opening and closing it). Thus, the subject received a reward no matter what she chose during the test phase, excluding the possibility of learning effects over the course of the 8 (children)/24 (chimpanzees) trials. Therefore, after the competitor’s turn, both sticks were still present when the subject chose; however, if the subject understood that one looked bigger to the competitor than the other, she should avoid the seemingly bigger, riskier stick in the social condition, but not in the nonsocial condition.

#### Data scoring and analysis

We scored a choice when the subject opened the sliding door to the left or right side (test). All choices were live coded, but trials were also videotaped for later analysis. A second independent observer coded a random sample of 20 % of all the sessions for reliability. The interrater agreement was excellent (Cohen’s κ = 1.0, *p* < .0001).

### Results

#### Procedure training and pretest

Two chimpanzees were excluded due to uncooperativeness (Annett, Corry). The others needed 2–3 procedure training sessions to pass the criterion [*M* (range) = 2, (2–3)], and all passed their session pretests [*M* = 99 % correct, (81–100)]. Children also did well in their pretests [*M* = 93 % correct, (75–100)].

#### Training

When choosing between a small and a big stick on a transparent barrier, both chimpanzees and children picked the big stick at high rates in the nonsocial condition [chimpanzees: *M* = 91 % (69–100) of trials with choice of bigger stick; children: *M* = 87 % (50–100)], children were quite successful in refraining from choosing the big stick after the competitor had chosen [*M* = 28 % (0–62)]. In contrast, chimpanzees still picked the location of the big stick (which was empty after the competitor had chosen) in more than half of the trials [*M* = 60 % (25–94)]. This preference was significantly above chance [one-sample *t* test comparing to 50 %: *t*(15) = 2.60; *p* = .02]. Both chimpanzees and children chose the big stick significantly more often when they were alone compared to when a competitor chose before them [repeated-measures ANOVA with order as between-subject factor; chimpanzees: *F*(1,14) = 32.64, *p* < .001, *η*^*2*^ = .70; children: *F*(1,14) = 100.92, *p* < .001, *η*^*2*^ = .88].

#### Test

**Fig. 3:**
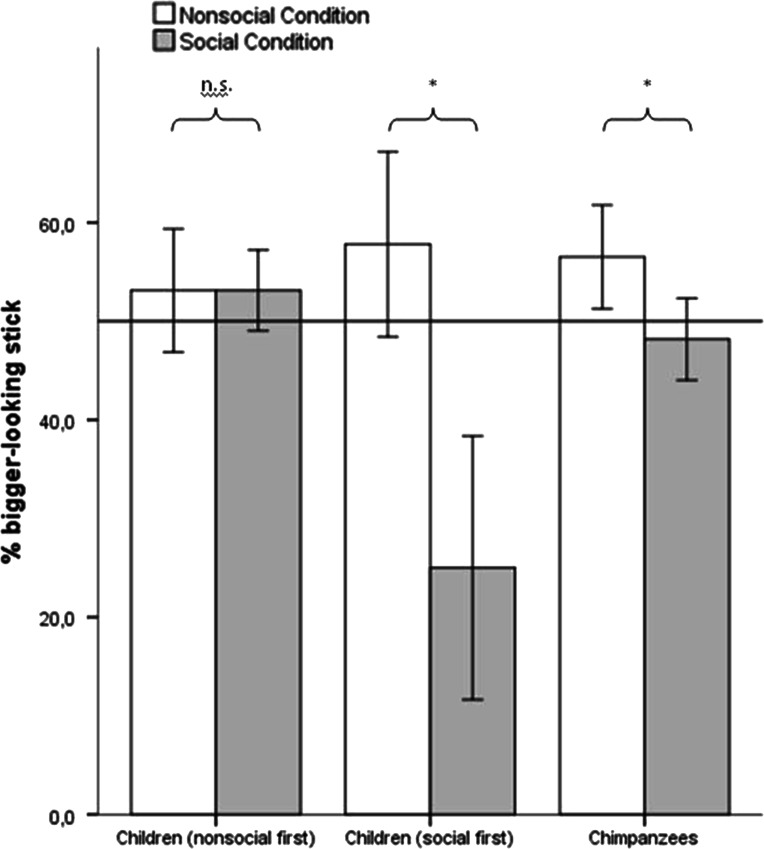
Percentage of trials in which the stick chosen looked bigger from the competitor’s side in the test conditions. Children’s performance is split for the order in which they received the conditions. Children who received the social condition first chose the smaller-looking stick significantly more in the social condition compared to the nonsocial control, while children in the nonsocial first group did not behave differently in the two conditions. *Error bars* indicate 95 % confidence intervals. The* horizontal line* indicates 50 %

Figure 3 shows that chimpanzees chose the stick that looked bigger to the competitor less often when the competitor was present, compared to the nonsocial control [repeated-measures ANOVA with order as between-subject factor: *F*(1,14) = 10.18, *p* = .007, *η*^*2*^ = .42; observed power = .843].

For children, we found an interaction between condition and order, *F*(1, 14) = 11.39, *p* = .005, *η*^*2*^ = .45 (see Fig. 3). Children who received the social condition first chose the smaller-looking stick significantly more in the social condition [*M* = 75 % (50–100)] compared to the nonsocial control [*M* = 43 % (13–50); paired-samples *t* test, *t*(7) = 2.97, *p* = .021], while children in the nonsocial first group did not behave differently in the two conditions [paired-samples *t* test, *t*(7) = .68, *p* = .52]: they chose the smaller-looking stick on an average of 47 % (38–63) (nonsocial condition) and 48.4 % (38–50) (social condition) of the trials, thus almost equally often in both conditions. Children’s performance did not differ between order groups in the nonsocial condition [independent samples *t* test: *t*(14) = .91, *p* = .38], but children with the social condition first performed significantly better in the social condition than children in the other group [independent samples *t* test: *t*(9) = 2.54, *p* = .032].

A majority of chimpanzees had a significant side preference when opening the sliding door: six out of 16 showed a side preference in both conditions, three others only in the social condition, and three others only in the nonsocial control.

In children, side preferences were less prominent. Out of the 16 children, two showed a side preference only in the social condition, two others only in the nonsocial control, and two others in both conditions.

We were also interested in whether subjects’ behavior differed from chance. We thus performed one-sample *t* tests and found that children behaved different from chance in the social condition [*t*(15) = 2.21, *p* = .043], but chose randomly in the nonsocial control [*t*(15) = 1.96, *p* = .07]. This was different for chimpanzees: They had a significant preference for the stick that looked bigger from the other side in the nonsocial control [*t*(15) = 2.47, *p* = .026], and this preference was reduced to chance when the competitor was present [*t*(15) = .88, *p* = .40].

We looked at individual performances to find out whether our effect is driven by a couple of successful individuals rather than the majority of subjects.

In the nonsocial control, two of the 16 chimpanzees had a significant preference for the bigger-looking stick (Pia, 18/24, and Frodo, 19/24). In the social condition, one chimpanzee had a significant preference for the correct (smaller-looking) stick (Alex, 17/24). The clearest difference in choice behavior between conditions was observed in Pia, who had no preference for any stick when she was alone (12/24), but avoided the bigger-looking stick significantly when a competitor had chosen before her. Altogether, 11 subjects chose the bigger-looking stick more when they were alone compared to choosing after a competitor, three showed no difference between conditions, and only two the reversed pattern.

Of all children in the nonsocial condition, only one individual had a significant preference for the bigger-looking stick (Julia, 7/8), while in the social condition, four individuals significantly preferred the correct (smaller-looking) stick (Oskar, Julia, Yannick: 7/8; Theophil, 8/8). The highest difference between conditions was observed in Julia, who chose six bigger-looking sticks more when she was alone compared to when a competitor had chosen before her.

### Discussion

When confronted with a competitor who possesses a mistaken perspective on two same-sized food sticks, chimpanzees and the group of children who received the social condition first correctly adjusted their behavior compared to a nonsocial control condition: Although they could see that both sticks had the same size, they avoided the seemingly bigger stick more often when choosing after the competitor than when no competitor was present. However, chimpanzees and children differed in an important way: While children chose randomly in the nonsocial control and avoided the seemingly bigger stick in the social condition, chimpanzees preferentially chose the stick that looked bigger from the other side in the nonsocial control but chose randomly in the social condition.

For children in the social first group, it is therefore clear that they perceived both sticks as equally big, and still understood the competitor’s mistaken perspective on the sticks. They thus engaged in Level 2 perspective-taking sensu Flavell et al. (1978, 1981). The fact that there was no difference between conditions if they received the nonsocial condition first could be due to a reduced motivation to participate in the study again after the first nonsocial session (which was clearly less entertaining than the social game), or the impression that they would receive a reward no matter what they chose. Children’s willingness to engage in Level 2 perspective-taking might thus be readily substituted by simpler and less demanding behavioral rules.

In contrast to children, the interpretation for the chimpanzees with regard to Level 2 perspective-taking is not as clear. Although they also significantly avoided the stick that looked bigger from the competitor’s side more in the social than in the nonsocial condition (as children did), this difference between conditions was less prominent compared to children, and they had a preference for this riskier stick in the nonsocial control. This preference might have been because the subjects perceived the stick further up as bigger and/or more attractive. There are at least two possible interpretations for the difference in their behavior between conditions. One is that chimpanzees, like children, really understood the competitor’s mistaken perspective, correctly predicted her behavior based on that (Level 2 perspective-taking) and overcame their own preference in the social condition. The other possibility is that they projected their preference of the stick further up to the competitor and consequently avoided this stick when she had chosen before them. This would mean that they understood the competitor’s motivation, but not that the competitor’s motivation was based on a false perception.

An alternative explanation would be that chimpanzees had a preference for the bigger-looking stick themselves and did not project this preference, but just got confused about the location of that stick as soon as a competitor was present. However, this is unlikely because they did not choose randomly in the competitor’s presence during the training in which they need similar memory skills.

One possible explanation for chimpanzees’ preference in the nonsocial control is that they perceived the stick further up as bigger. This preference could have arisen, e.g., by the chimpanzees being primed to perceive one stick as bigger than the other in the training. Previous studies have shown that chimpanzees and other nonhuman primates are susceptible to misleading visual input, but often to a different degree compared to humans (Barbet and Fagot 2002, 2007; Fujita 1997, 2001; Parron and Fagot 2007; Suganuma et al. 2007). Chimpanzees could also have perceived the stick further up as more attractive for other reasons, e.g., as easier to grab.

We thus conducted Study 2 with chimpanzees to discriminate between the Level 2 and the preference projection interpretations by changing the setup such that a preference for one of the sticks in the nonsocial control was less likely.

## Study 2: chimpanzees—a follow-up

### Method

#### Subjects

Sixteen chimpanzees (*Pan troglodytes*) participated in this experiment, nine females and seven males ranging in age from 12 to 22 years (*M* = 15.4 years). One chimpanzee was excluded due to uncooperativeness (Pasa, 14) and four others because they did not reach criterion in the pretest within 10 sessions (Kalema, 17; Kisembo, 14; Nani, 12; Sally, 22). In addition, one chimpanzee served as competitor (Asega; male, 15).

All subjects were living at Ngamba Island Chimpanzee Sanctuary, Uganda (www.ngambaisland.org). All came to the sanctuary as orphans as a result of the illegal bushmeat trade and were then raised by humans together with peers and later often adopted by conspecific foster mothers. They all lived in social groups at the time of testing and could move freely in a 100-acre rainforest during the day. They moved voluntarily into their enclosure for eating and sleeping in the evenings, where they were tested in the mornings before moving into the forest. They were fed according to their daily routine and never food- or water-deprived. Subjects all had previous experience with participating in experimental studies.

#### Materials

**Fig. 4:**
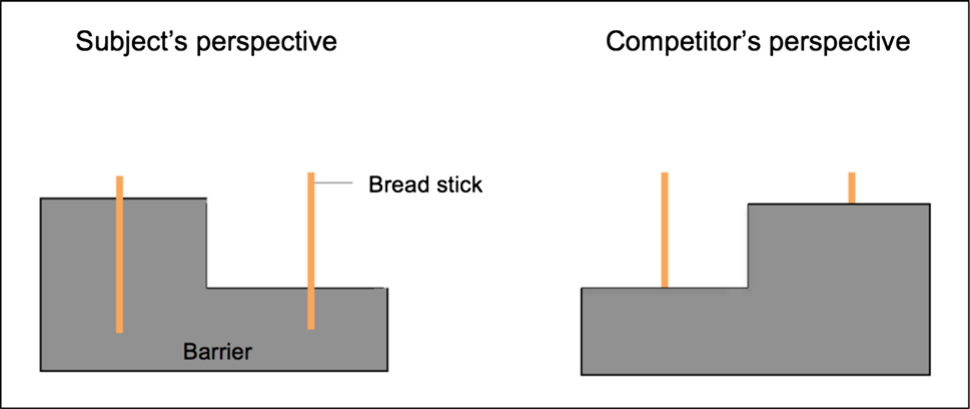
Subject’s and competitor’s view of the experimental setup in Study 2. While the subject could see that both sticks had the same size, to the competitor one stick seemed bigger than the other

The setup was the same as in Study 1, with the only difference that we substituted the opaque barrier to which the sticks were attached with a barrier that was less likely to create a stick preference for the subjects because both sticks were at the same height (Fig. 4). The barrier was 44 × 18 cm, but with a 22 × 6 cm piece removed.

#### Design

The design was the same as in Study 1. Five chimpanzees received the nonsocial condition first, the other six the social condition first. The order of conditions was again counterbalanced for age and sex.

#### Procedure, data scoring, and analysis

All as in Study 1. The interobserver agreement was again excellent (Cohen’s κ = 1.0, *p* < .0001).

### Results

#### Procedure training and pretest

Chimpanzees needed on average seven (2–11) door training sessions to pass the criterion, and all passed their session pretests [*M* = 87 % correct, (75–100)].

#### Training

When choosing between a small and a big stick on a transparent barrier, chimpanzees picked the big stick at high rates in the nonsocial condition [*M* = 71 % (56–81)], but inhibited going for the big stick after the competitor had chosen [*M* = 45 % (25–63)]. They chose the big stick significantly more often when they were alone compared to when a competitor chose before them [repeated-measures ANOVA with order as between-subject factor; *F*(1,9) = 38.98, *p* < .001, *η*^*2*^ = .81]. Their behavior in the social condition was not significantly different from chance [one-sample *t* test comparing to 50 %: *t*(10) = 1.70; *p* = .12].

#### Test

**Fig. 5:**
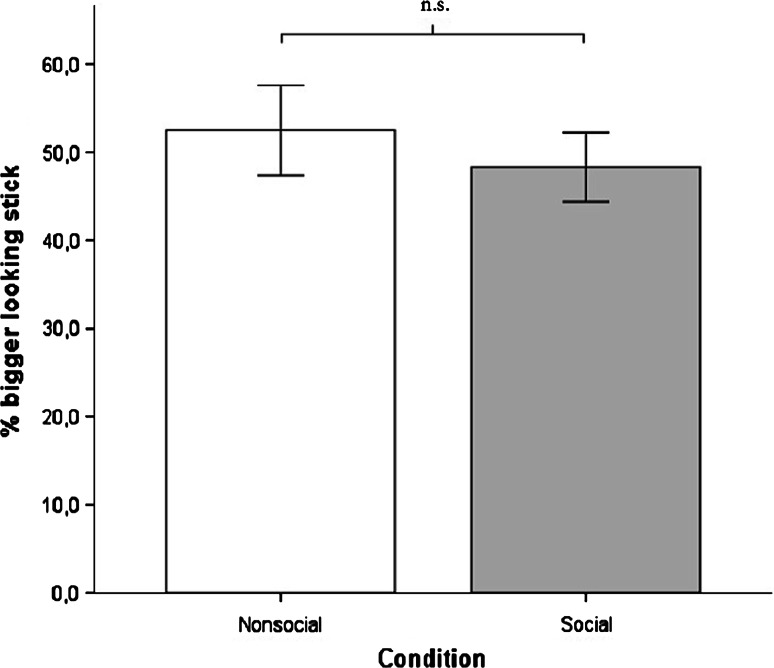
Percentage of trials in which the stick was chosen that looked bigger from the competitor’s side in Study 2. *Error bars* indicate 95 % confidence intervals

Figure 5 shows that chimpanzees did not choose the stick that looked bigger to the competitor less often when the competitor was present, compared to the nonsocial control [repeated-measures ANOVA with order as between-subject factor; *F*(1,9) = 1.58, *p* = .24, *η*^*2*^ = .15], and chose in both conditions randomly. Seven out of 11 individuals had a significant side preference in both conditions, two only in the social condition, and one only in the nonsocial control. None of the individuals had a significant preference for the bigger-looking or smaller-looking stick in any condition.

### Discussion

In contrast to Study 1, chimpanzees now did not prefer one of the sticks in the nonsocial condition any more. However, they were now also not able to correctly predict the behavior of a competitor who was holding a mistaken perspective on the sticks.

While this indicated that we successfully eliminated the stick preference in the nonsocial condition, we also eliminated their avoidance of the riskier stick in the social condition. What could account for this result?

First, this could be evidence that chimpanzees, when they do not have a preference themselves, are not able to understand others’ preferences. This would be in line with research on false-belief understanding, in which chimpanzees consistently fail to attribute false beliefs, thus representations that differ from their own, to others (Call and Tomasello 1999; Kaminski et al. 2008; Krachun et al. 2009, 2010; O’Connell and Dunbar 2003).

Second, it is possible that with the changed setup, it was more difficult for chimpanzees to reason about the stick size relations from the competitor’s perspective. Although we tried to change the barrier as little as possible to make the apparent differences in stick attractiveness disappear and kept the procedure exactly the same, we cannot fully rule out this concern.

Third, we tested a different sample of chimpanzees that might differ in their cognitive capacities to understand others’ perspectives and their ability to play this competitive game. In addition, the competitor was male in Study 2 and female in Study 1, and hierarchy processes could have played a role. Two major points speak against this explanation. First, the sample in Study 2 was more successful in the training compared to the sample in Study 1, an indicator for their even better understanding of the rules of the game and for their willingness to compete also with a male conspecific. Second, there are several studies with this back-and-forth paradigm that have successfully been conducted with the chimpanzees in sample 2 (J. Kaminski, unpublished data). In addition, both samples had previous experience with experimental testing. We thus think that it is unlikely that they failed because of mere confusion about the game, a lack of cognitive capacities or aloofness with a male competitor.

## General discussion

We conducted two studies to assess whether chimpanzees can correctly predict a competitor’s behavior that is based on a mistaken perspective. In Study 1, we found that chimpanzees as well as the group of 6-year-old children who received the social condition first avoided the food stick (out of two same-sized sticks) that looked longer from the competitor’s side more when a competitor had chosen before them compared to when they were alone.

While it was clear from the nonsocial control that child subjects correctly perceived the sticks as having the same length themselves, chimpanzees perceived the stick that looked bigger to the competitor as more attractive themselves. They could thus have solved the task either like children by Level 2 perspective-taking, or by projecting their own preference to the other. To distinguish between these two possibilities, we carried out a second study with a slight change in the setup that eliminated the perceived difference in the sticks’ attractiveness. With this new setup, chimpanzees did not show any differences in their choice behavior between social and nonsocial condition any more.

Our results suggest that chimpanzees successfully predicted what the competitor would choose when they were guided by their own preference. However, when they did not have a preference themselves and they were required to understand the other’s mistaken perspective, they failed to do so. In contrast, 6-year-old children successfully contrasted their own true perspective with the mistaken perspective of the competitor, thus engaged in Level 2 perspective-taking (Flavell et al. 1978, 1981). As all of our test trials were nondifferentially rewarded, we can exclude learning as an explanation for these results.

Previous research has shown that children successfully master most Level 2 perspective-taking tasks by the age of 4–5 years (Flavell et al. 1980, 1981; Masangkay et al. 1974; Pillow and Flavell 1986). This parallels the development of explicit false-belief reasoning, a skill that unfolds around the same time (Wellman et al. 2001; Wimmer and Perner 1983). This parallel development might not be a coincidence—both require the skill of contrasting differing representations (see Flavell 1986; Gopnik and Astington 1988). In fact, results from neuroscience support this claim: Aichhorn et al. (2006) found that false belief and higher-level visual perspective-taking tasks partially activate the same brain areas.

Chimpanzees seem to have problems with contrasting perspectives. In various studies that test their false-belief understanding, chimpanzees consistently fail to do so (Call and Tomasello 1999; Kaminski et al. 2008; Krachun et al. 2009, 2010; O’Connell and Dunbar 2003). They also fail to create misleading information by actively hiding food from competitors (Karg et al. 2015a, b). However, they successfully attribute knowledge and ignorance to others (Crockford et al. 2012; Hare et al. 2000, 2001; Kaminski et al. 2008), a skill that could underlie their success in Level 1 perspective-taking tasks.

But chimpanzees did not fail entirely in our experiment—when they themselves had a preference, they were able to use it to correctly predict the competitor’s choice. While there is no evidence for Level 2 perspective-taking, it still indicates two remarkable skills. First, chimpanzees are able to overcome the seduction of choosing the item that they prefer themselves when they choose after a competitor, supporting two recent findings by Schmelz et al. (2011, 2013). This is a noteworthy finding given that chimpanzees have only limited inhibitory control (see, e.g., Boysen 1996; Vlamings et al. 2006, 2010). The difficulty to inhibit choosing the bigger item decreases with the ratio of the presented food quantities (Boysen et al. 2001; Uher and Call 2008). As there were no real size differences between the sticks from the subject’s perspective, the perceived difference in size or attractiveness was probably small and easy to overcome.

Second, our results indicate that chimpanzees understand others’ desires, at least if they match their own. Buttelmann et al. (2009) found that chimpanzees used others’ facial expression (happiness/disgust) when choosing containers with food inside. However, considering that chimpanzees have problems contrasting conflicting perspectives, it is an open question whether they can correctly predict another’s preference if it does not match their own.

But if chimpanzees really have trouble contrasting differing perspectives, why do they succeed in appearance–reality tasks, in which they have to contrast their apparent actual perspective with reality? There is a crucial difference between these tasks that could explain this difference. In our Level 2 perspective-taking task, subjects had no experience with the other’s perspective, while in the appearance–reality tasks (Karg et al. 2014; Krachun and Call 2009) they have experienced the true perspective before. This self-experience could help them to understand the differing perspective as the representation can be retrieved from their memory. Another recent study supports this view. Karg et al. (2015a, b) found that chimpanzees understood that a competitor could see through a screen while they themselves could not, after having gained visual self-experience with the other perspective on the screen before. The role of self-experience in understanding other perspectives is further supported by a study with scrub jays (Clayton et al. 2007). Scrub jays who had pilfered other jays’ caches before re-cached their food when the hiding event was observed by a competitor; naïve jays without pilfering experience did not do so. It is thus an interesting question whether experiencing the setup from the competitor’s side first may facilitate the subjects’ success in perspective-taking tasks.

In sum, while there is evidence that chimpanzees are skilled in complex Level 1 perspective-taking and can use their own experience to reason about the perspectives and preferences of others, ‘putting themselves in the mental shoes’ of others that are really different seems to be a cognitive challenge for them. However, the perspective-taking process might be facilitated by own experience with the alien perspective or preference.

## References

[CR1] Aichhorn M, Perner J, Kronbichler M, Staffen W, Ladurner G (2006). Do visual perspective tasks need theory of mind?. Neuroimage.

[CR2] Anderson M, Mitchell RW, Thompson NS (1986). Cultural concatenation of deceit and secrecy. Deception: perspectives on human and nonhuman deceit.

[CR3] Baillargeon R, Scott RM, He Z (2010). False-belief understanding in infants. Trends Cogn Sci.

[CR4] Barbet I, Fagot J (2002). Perception of the corridor illusion by baboons (*Papio papio*). Behav Brain Res.

[CR5] Barbet I, Fagot J (2007). Control of the corridor illusion in baboons (*Papio papio*) by Gradient and linear perspective depth cues. Perception.

[CR6] Boysen ST, Russon AE, Bard KA, Parker ST (1996). “More is less”: the elicitation of rule-governed resource distribution in chimpanzees. Reaching into thought: the minds of the great apes.

[CR7] Boysen ST, Berntson GG, Mukobi KL (2001). Size matters: impact of item size and quantity on array choice by chimpanzees (*Pan troglodytes*). J Comp Psychol.

[CR8] Bräuer J, Call J, Tomasello M (2005). All great ape species follow gaze to distant locations and around barriers. J Comp Psychol.

[CR9] Bräuer J, Call J, Tomasello M (2007). Chimpanzees really know what others can see in a competitive situation. Anim Cogn.

[CR10] Buttelmann D, Call J, Tomasello M (2009). Do great apes use emotional expressions to infer desires?. Dev Sci.

[CR11] Call J, Tomasello M (1999). A nonverbal false belief task: the performance of children and great apes. Child Dev.

[CR12] Clayton NS, Dally JM, Emery NJ (2007). Social cognition by food-caching corvids. The western scrub-jay as a natural psychologist. Philos Trans R Soc Lond B.

[CR13] Crockford C, Wittig RM, Mundry R, Zuberbühler K (2012). Wild chimpanzees inform ignorant group members of danger. Curr Biol.

[CR14] de Waal FBM (1998). Chimpanzee politics: power and sex among apes.

[CR15] Flavell JH (1986). The development of children’s knowledge about the appearance–reality distinction. Am Psychol.

[CR16] Flavell JH, Shipstead SG, Croft K (1978). Young children’s knowledge about visual perception: hiding objects from others. Child Dev.

[CR17] Flavell JH, Flavell ER, Green FL, Wilcox SA (1980). Young childrens knowledge about visual-perception: effect of observers distance from target on perceptual clarity of target. Dev Psychol.

[CR18] Flavell JH, Everett BA, Croft K, Flavell ER (1981). Young children’s knowledge about visual perception: further evidence for the level 1 level 2 distinction. Dev Psychol.

[CR19] Fujita K (1997). Perception of the Ponzo illusion by rhesus monkeys, chimpanzees, and humans: similarity and difference in the three primate species. Percept Psychophys.

[CR20] Fujita K, Matsuzawa T (2001). What you see is different from what i see: species differences in visual perception. Primate origins of human cognition and behavior.

[CR21] Gopnik A, Astington JW (1988). Children’s understanding of representational change and its relation to the understanding of false belief and the appearance–reality distinction. Child Dev.

[CR22] Hare B, Call J, Agnetta B, Tomasello M (2000). Chimpanzees know what conspecifics do and do not see. Anim Behav.

[CR23] Hare B, Call J, Tomasello M (2001). Do chimpanzees know what conspecifics know?. Anim Behav.

[CR24] Hare B, Call J, Tomasello M (2006). Chimpanzees deceive a human competitor by hiding. Cognition.

[CR25] Heyes CM (1993). Anecdotes, training, trapping and triangulating: do animals attribute mental states?. Anim Behav.

[CR26] Heyes CM (1998). Theory of mind in nonhuman primates. Behav Brain Sci.

[CR27] Kaminski J, Call J, Tomasello M (2008). Chimpanzees know what others know, but not what they believe. Cognition.

[CR28] Karg K, Schmelz M, Call J, Tomasello M (2014). All great ape species (*Gorilla gorilla*, *Pan paniscus*, *Pan troglodytes*, *Pongo abelii*) and two-and-a-half-year-old children (*Homo sapiens*) discriminate appearance from reality. J Comp Psychol.

[CR29] Karg K, Schmelz M, Call J, Tomasello M (2015). The goggles experiment: can chimpanzees use self-experience to infer what a competitor can see?. Anim Behav.

[CR30] Karg K, Schmelz M, Call J, Tomasello M (2015). Chimpanzees strategically manipulate what others can see. Anim Cogn.

[CR31] Krachun C, Call J (2009). Chimpanzees (*Pan troglodytes*) know what can be seen from where. Anim Cogn.

[CR32] Krachun C, Carpenter M, Call J, Tomasello M (2009). A competitive nonverbal false belief task for children and apes. Dev Sci.

[CR33] Krachun C, Carpenter M, Call J, Tomasello M (2010). A new change-of-contents false belief test: children and chimpanzees compared. Int J Comp Psychol.

[CR34] MacLean EL, Hare B (2012). Bonobos and chimpanzees infer the target of another’s attention. Anim Behav.

[CR35] Masangkay ZS, McCluskey KA, McIntyre CW, Sims-Knight J, Vaughn BE, Flavell JH (1974). The early development of inferences about the visual percepts of others. Child Dev.

[CR36] McGuigan N, Doherty MJ (2002). The relation between hiding skill and judgment of eye direction in preschool children. Dev Psychol.

[CR37] Melis AP, Call J, Tomasello M (2006). Chimpanzees (*Pan troglodytes*) conceal visual and auditory information from others. J Comp Psychol.

[CR38] Moll H, Meltzoff AN (2011). How does it look? Level 2 perspective-taking at 36 months of age. Child Dev.

[CR39] Moll H, Tomasello M (2006). Level I perspective-taking at 24 months of age. Br J Dev Psychol.

[CR40] O’Connell SM, Dunbar RIM (2003). A test for comprehension of false belief in chimpanzees. Evol Cogn.

[CR41] Parron C, Fagot J (2007). Comparison of grouping abilities in humans (*Homo sapiens*) and baboons (*Papio papio*) with the Ebbinghaus illusion. J Comp Psychol.

[CR42] Pillow BH, Flavell JH (1986). Young childrens knowledge about visual-perception: projective size and shape. Child Dev.

[CR43] Schmelz M, Call J, Tomasello M (2011). Chimpanzees know that others make inferences. Proc Natl Acad Sci USA.

[CR44] Schmelz M, Call J, Tomasello M (2013). Chimpanzees predict that a competitor’s preference will match their own. Biol Lett.

[CR45] Suganuma E, Pessoa VF, Monge-Fuentes V, Castro BM, Tavares MCH (2007). Perception of the Müller–Lyer illusion in capuchin monkeys (*Cebus apella*). Behav Brain Res.

[CR46] Tomasello M, Call J, Hare B (1998). Five primate species follow the visual gaze of conspecifics. Anim Behav.

[CR47] Uher J, Call J (2008). How the great apes (*Pan troglodytes*, *Pongo pygmaeus*, *Pan paniscus*, *Gorilla gorilla*) perform on the reversed reward contingency task ii: transfer to new quantities, long-term retention, and the impact of quantity ratios. J Comp Psychol.

[CR48] Vlamings PH, Uher J, Call J (2006). How the great apes (*Pan troglodytes*, *Pongo pygmaeus*, *Pan paniscus*, and *Gorilla gorilla*) perform on the reversed contingency task: the effects of food quantity and food visibility. J Exp Psychol Anim B.

[CR49] Vlamings PH, Hare B, Call J (2010). Reaching around barriers: the performance of the great apes and 3–5-year-old children. Anim Cogn.

[CR50] Wellman HM, Cross D, Watson J (2001). Meta-analysis of theory-of-mind development: the truth about false belief. Child Dev.

[CR51] Whiten A, Byrne RW (1988). Tactical deception in primates. Behav Brain Sci.

[CR52] Wimmer H, Perner J (1983). Beliefs about beliefs: representation and constraining function of wrong beliefs in young children’s understanding of deception. Cognition.

